# A Twice Electrochemical-Etching Method to Fabricate Superhydrophobic-Superhydrophilic Patterns for Biomimetic Fog Harvest

**DOI:** 10.1038/s41598-017-09108-1

**Published:** 2017-08-18

**Authors:** Xiaolong Yang, Jinlong Song, Junkai Liu, Xin Liu, Zhuji Jin

**Affiliations:** 1 Key Laboratory for Precision and Non-traditional Machining Technology of the Ministry of Education, Dalian University of Technology, Dalian, 116024 P. R. China; 2 Collaborative Innovation Center of Major Machine Manufacturing in Liaoning, Dalian University of Technology, Dalian, 116024 P. R. China

## Abstract

Superhydrophobic-superhydrophilic patterned surfaces have attracted more and more attention due to their great potential applications in the fog harvest process. In this work, we developed a simple and universal electrochemical-etching method to fabricate the superhydrophobic-superhydrophilic patterned surface on metal superhydrophobic substrates. The anti-electrochemical corrosion property of superhydrophobic substrates and the dependence of electrochemical etching potential on the wettability of the fabricated dimples were investigated on Al samples. Results showed that high etching potential was beneficial for efficiently producing a uniform superhydrophilic dimple. Fabrication of long-term superhydrophilic dimples on the Al superhydrophobic substrate was achieved by combining the masked electrochemical etching and boiling-water immersion methods. A long-term wedge-shaped superhydrophilic dimple array was fabricated on a superhydrophobic surface. The fog harvest test showed that the surface with a wedge-shaped pattern array had high water collection efficiency. Condensing water on the pattern was easy to converge and depart due to the internal Laplace pressure gradient of the liquid and the contact angle hysteresis contrast on the surface. The Furmidge equation was applied to explain the droplet departing mechanism and to control the departing volume. The fabrication technique and research of the fog harvest process may guide the design of new water collection devices.

## Introduction

Superhydrophobic surfaces are biomimetic surfaces with contact angles (CAs) higher than 150° ^1^ and have been intensely investigated in different domains due to their wide applications in various technical areas such as self-cleaning^2–4^, anti-icing^5–9^, anti-corrosion^10, 11^, drag reduction^12–14^ and oil/water separation^15–19^. Among them, superhydrophobic surfaces with hydrophilic/superhydrophilic patterns have attracted more and more attention due to a variety of potential applications like fog harvesting devices^20–29^ and other droplet manipulation components^30–43^. To date, various methods including plasma treatment^24, 28, 44, 45^, laser irradiation^37, 46–50^, micromilling^35, 43, 51^, lithography^52, 53^, soft lithography^21^, UV-driven transition^20, 31, 54, 55^ and printing technology^23, 33, 36, 56^ have been developed to obtain such superhydrophobic-hydrophilic/superhydrophilic surfaces. For instance, Franssila *et al*.^44^ reported a method combining plasma deposition, lithography and oxygen plasma treatment to prepare silicon nanograss superhydrophobic surfaces with superhydrophilic patterns. Lai *et al*.^48^ fabricated high-accuracy superhydrophilic patterns on a superhydrophobic TiO_2_ nanotube array (TNA) surface using a femtosecond laser. The dimension and morphology of the patterns were easily controlled by adjusting the output power energy and scanning rate. You *et al*.^53^ successfully constructed micro-scale superhydrophilic tracks on anodic aluminum oxide surface by combining lithography and dopamine coating technology. They realized the precisely controlled droplet movement in an energy-efficient manner. Lee *et al*.^21^ prepared polydopamine-strip-patterned superhydrophobic surfaces using a PDMS mold and water droplets were transported directly along the line patterns. Liu *et al*.^45^ altered a fluoroalkylsilane modified superhydrophobic surface to achieve superhydrophilicity by using atmospheric pressure plasma treatment; Superhydrophilic patterns with different shapes could be obtained when masks were applied in the treatment. Zheng *et al*.^20^ took advantage of heptadecafluorodecyl-trimethoxysilane’s (FAS) photocatalytic decomposition property and prepared superhydrophilic patterns with various shapes on the FAS modified TNA surfaces by masked illumination of UV light. Guo *et al*.^22^ prepared superhydrophilic surfaces with circle superhydrophobic patterns by site-selectively modifying superhydrophilic Cu(OH)_2_ nano-needle structures with n-octadecylthiol (ODT)/ethanol solution. These fabricated patterned surfaces were macroscopically similar to a desert beetle’s back surface showing superior water harvest efficiency. Wang *et al*.^23^ employed an inkjet printer to directly print superhydrophilic dopamine solution onto the prepared superhydrophobic substrate. The produced micropatterned surface, inspired by the desert beetle, exhibited enhanced water collection ability. The technologies mentioned above can prepare superhydrophilic patterns with various shapes and dimensions on various superhydrophobic substrates. Nevertheless, superhydrophilic patterns fabricated by plasma treatment suffer from the aging issue that the superhydrophilicity would expire after exposure for hours in the ambient environment^57^. UV-driven transition is effective for limited coatings and laser irradiation is dependent on expensive equipment, both of which hinder wide industrial promotion. And so far, to the best of our knowledge, few researchers have developed an inexpensive, easy-to-implement and universal technique that can fabricate superhydrophobic-superhydrophilic patterns on multiple kinds of metal substrates.

Electrochemical-etching, a very commonly used non-traditional machining technology has been employed to process diverse metal materials^58–61^ and when combined with masking technology, it can also fabricate structures with various patterns^62^. In this work, a twice electrochemical-etching method was proposed for the first time to fabricate superhydrophobic-superhydrophilic patterned surface on Al substrates. Electrochemical-etching and surface chemical modification technologies were first utilized to prepare superhydrophobic substrates. Then the electrochemical-etching technology was applied with a mask to directly fabricate superhydrophilic dimples on the prepared superhydrophobic surfaces (see Supplementary Information Fig. S1). The influence of the second etching parameters such as etching voltages and etching time on the wettability of the fabricated dimple was investigated. By immersing the second etched substrate in the boiling water before mask removal, long-term superhydrophilicity of the dimples can be achieved.

Studies on fog harvest have attracted more attention in recent years^63^ due to the water shortages from the large world population. Through the use of patterning technology, different patterns were designed and fabricated on superhydrophobic Al substrates and fog harvest tests were carried out. Results showed that superhydrophobic surfaces with a wedge-shaped superhydrophilic dimple array had high water collection efficiency due to rapid condensate drainage caused by both the internal Laplace pressure gradient of the liquid on the wedge-shaped superhydrophilic domains and the CA hysteresis contrast on the surface. The proposed twice electrochemical etching method is universal and can be extended to different metal substrates, such as magnesium, titanium, etc. This new technology can possibly be applied in designing new categories of water collection devices by mimicking the desert beetle.

## Results and Discussion

### Anti-electrochemical corrosion of the superhydrophobic layer

**Figure 1:**
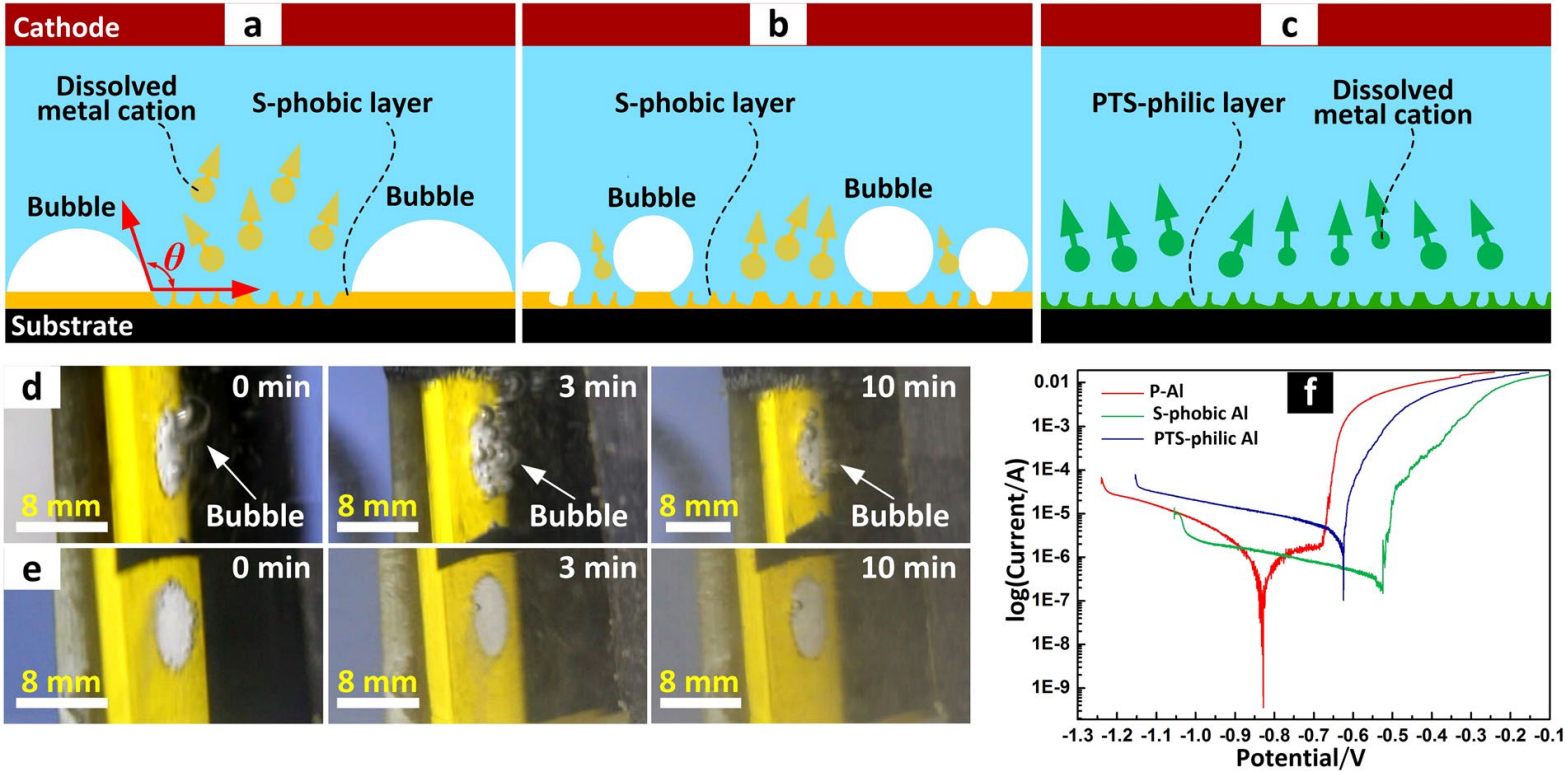
Etching process and polarization curve of different surfaces. Schematic illustrations show the etching process (**a**,**b**) on superhydrophobic Al area and (**c**) plasma-treated superhydrophilic area. The screenshots show the etching process (**d**) on superhydrophobic Al area and (**e**) plasma-treated superhydrophilic area. (**f**) Polarization curve of polished Al (P-Al), superhydrophobic Al (S-phobic Al) and plasma-treated superhydrophilic Al (PTS-philic Al), the electrolyte was 1 wt% NaCl aqueous solution.

The superhydrophobic layer is difficult to etch away at low voltage because of its high electrochemical corrosion potential. Water CA of superhydrophobic layer is above 150° which means long triple-phase contact line (TPCL) of the bubbles adhered on the superhydrophobic layer (Fig. 1a). It’s difficult for those bubbles to detach from the substrate due to the long TPCL. The bubbles act as a protective screen to prevent the substrate from making contact with the electrolyte and dissolving in the electrochemical etching process. The electrochemical etching process takes place in some areas where the CAs are not high enough to form a protective layer. With increasing etching time, the etched area expands lengthways, the bubble area gradually decreases and some isolated unetched regions thereupon form (Fig. 1b). At this stage, the bubble detaches from the etched region because of the decreasing TPCL (Fig. 1b). As the etched area expands, the isolated area would disappear and a uniform etched superhydrophilic layer is produced. On the other hand, a superhydrophilic layer with a CA below 10° causes the bubbles generated on the superhydrophilic surface to easily leave the surface, allowing the surface to fully contact the electrolyte, and therefore could make the etching process uniform and fast (Fig. 1c). Figure 1d and e are photo sequences showing the etching processes on a superhydrophobic surface and a plasma-treated superhydrophilic surface, respectively. Many bubbles were generated and adhered on the superhydrophobic layer during the initial etching stage while there were almost no bubbles shown on the plasma-treated superhydrophilic layer, which corresponds to the previous assumption. As shown in Fig. 1f, the generated bubbles made the electrochemical corrosion potential of the superhydrophobic layer higher than the plasma-treated superhydrophilic Al surface and a polished Al surface. Therefore, an electrochemical potential high enough to conquer the electrochemical corrosion of the superhydrophobic layer must be applied during the etching process. In order to produce a homogeneous superhydrophilic dimple using this twice etching method, the influence of the applied potential on the morphology and wettability of the etched dimples were investigated in the following section.

### Effect of etching potential on the dimple wettability

**Figure 2:**
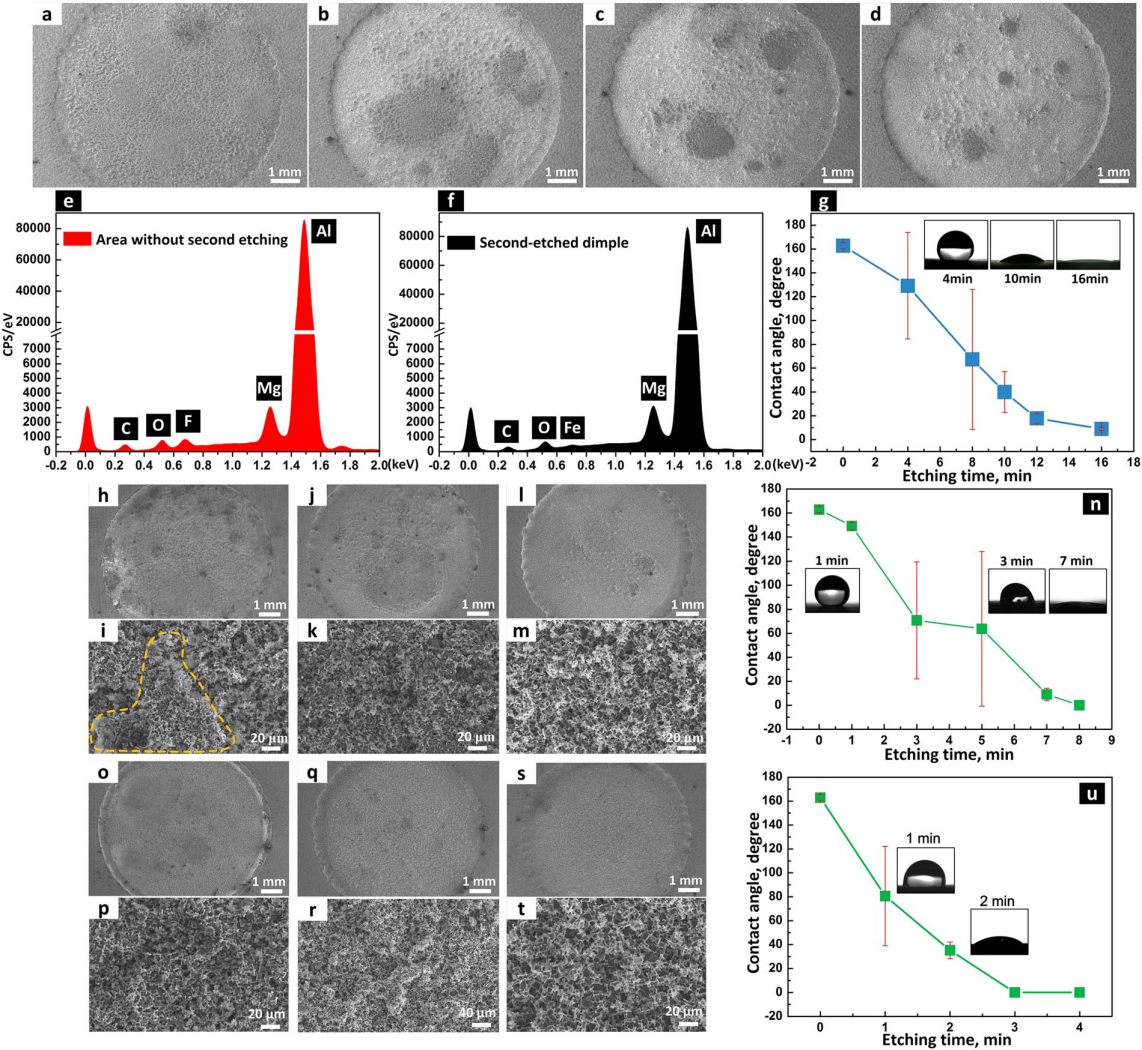
How morphology, surface elements and wettability changed when 10–30 V etching voltages were applied. SEM images of dimples that were electrochemically etched at 10 V for (**a**) 4, (**b**) 8, (**c**) 12 and (**d**) 16 minutes, respectively. EDS spectrums of (**e**) superhydrophobic area without second etching and (**f**) the second etched dimple area. (**g**) CAs of 5 μL water droplets in the dimples etched at 10 V for different times. SEM images of dimples that were electrochemically etched at 20 V for (**h**) 3, (**j**) 5 and (**l**) 8 minutes, respectively. (**i**) Magnified SEM image of the boundary between the unetched isolated area and the etched area in (**h**). (**k**) Magnified SEM image of the unetched isolated area in (**j**). (**m**) Magnified SEM image of the bulged area in (**l**). (**n**) CAs of 5 μL water droplets in the dimples etched at 20 V for different times. SEM images and magnified SEM images of dimples that were electrochemically etched for 30 V at (**o**,**p**) 1, (**q**,**r**) 2 and (**s**,**t**) 3 minutes, respectively. (**u**) CAs of 5 μL water droplets in the dimples etched at 30 V for different times.

Different etching voltages were applied in the second etching process (Fig. 2). Figure 2a–d show SEM images of dimples electrochemically etched at 10 V for different times. The EDS spectrum of the superhydrophobic layer detected the F element which corresponds to the -CF_3_ group of the self-assembled hydrophobic film (Fig. 2e)^64^. When an electrochemical potential was applied between the surface and the cathode, the superhydrophobic layer was gradually dissolved. Figure 2f shows the EDS spectrums of the etched area in the dimple. The absence of the F element indicates that the etching process removed the low-surface-energy -CF_3_ group. However, due to the high electrochemical corrosion resistance of the superhydrophobic layer, the 10 V etching voltage was not enough for a uniform dissolving process. Hence there appeared some isolated unetched areas in the etched dimple. The isolated areas and the average CA values on the dimples decreased with the increase of the etching time (Fig. 2d and g). Even though the CA of the obtained dimples was below 10° after 16 minute etching, some isolated areas remained with diameters up to 1.0 mm.

When a 20 V etching potential was applied, boundary between the isolated unetched area and the etched area was still not clear after 3 minute etching (Fig. 2h) due to low material removal. However, the boundary can be distinguished in the magnified SEM images of the dimple (Fig. 2i). With an increased etching time to 5 minutes, only one isolated area with a diameter of ~4 mm remained (Fig. 2j and k). After 8 minute etching, the isolated unetched area disappeared leaving behind some bulges with diameters of less than 1 mm in the middle of the dimple (Fig. 2l and m). At this state the dimple was superhydrophilic (Fig. 2n). Thus, the etching voltage of 20 V was enough to produce a uniform superhydrophilic dimple, but not enough to produce a morphologically flat dimple. Figure 2o–t exhibit SEM images of the dimples etched at 30 V for different times. Some isolated areas shown at the initial etching stage (Fig. 2o and p) decreased and disappeared almost completely at an etching time of 2 min (Fig. 2q and r). After a 3 minute etching process, a homogeneously flat superhydrophilic dimple with a diameter of ~8 mm was obtained (Fig. 2s–u).

**Figure 3:**
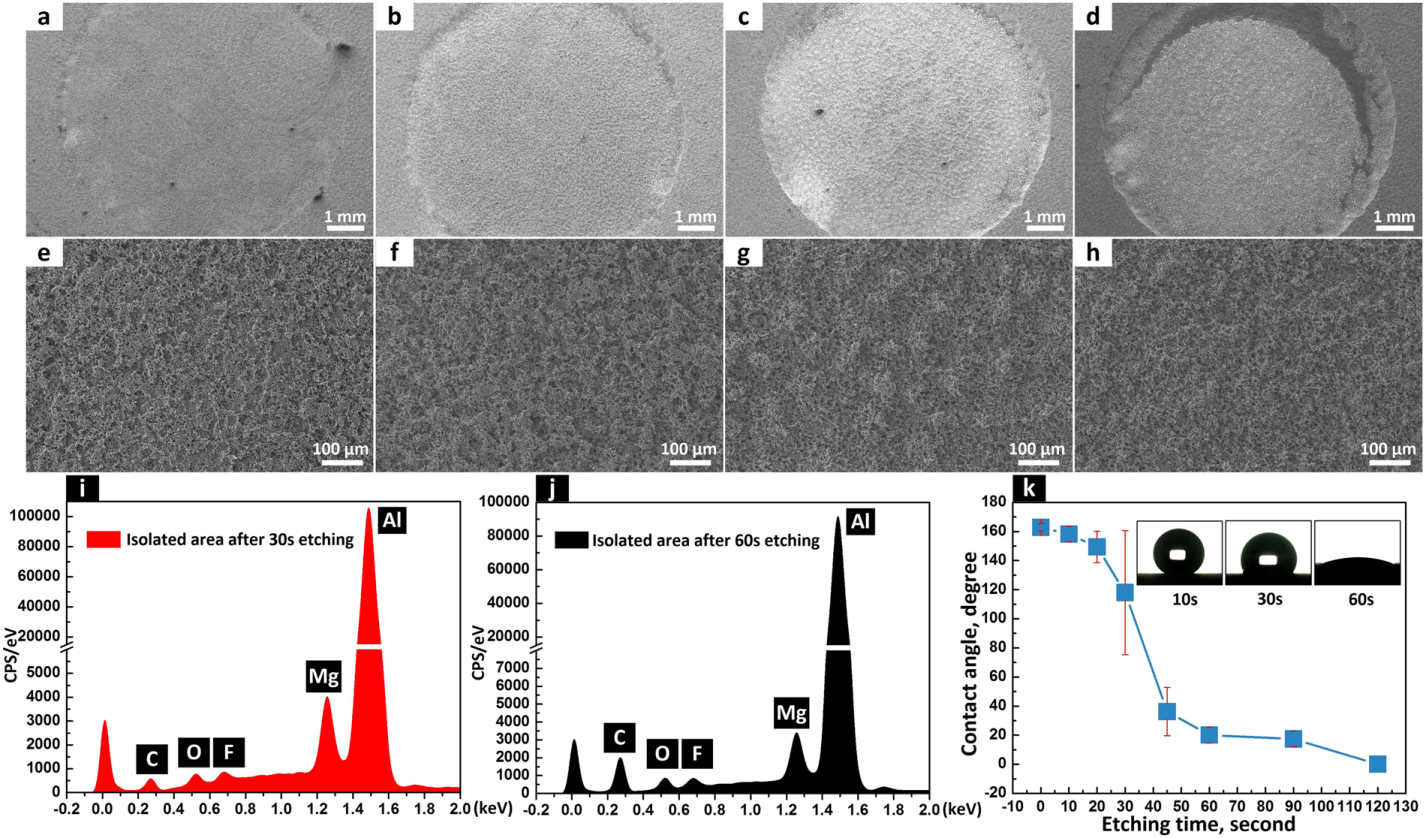
How morphology, surface elements and wettability changed when 60 V etching voltage was applied. SEM images and magnified SEM images of dimples that were electrochemically etched at 60 V for (**a**,**e**) 10, (**b**,**f**) 30, (**c**,**g**) 60 and (**d**,**h**) 120 seconds, respectively. EDS spectrums of the isolated area in the dimples after (**i**) 30 and (**j**) 60 seconds etching. (**k**) CAs of 5 μL water droplets in the dimples that were etched at 60 V for different times.

When the etching voltage was set to 60 V, the second etching process seemed uniform and no obvious isolated unetched areas appeared in the dimple (Fig. 3a–c). From the magnified SEM images numerous bulged areas with dimensions of hundred microns were connected with each other and distributed evenly in the dimple (Fig. 3e–g). The F element was detected in the EDS spectrums of the bulged area in the dimples after 30 second etching (Fig. 3i), which further indicated that the bulged area was not etched or not etched completely to remove the self-assembled hydrophobic film. With the etching time increased from 10 to 60 second, the etched areas increased and connected with neighboring ones while the bulged areas gradually decreased and became isolated. Even though the F element was detected on the isolated bulge in dimple after 60 second etching (Fig. 3j), the CA in the dimple had decreased to ~20° because of the low ratio of the isolated bulge to the etched area. When the etching time was increased to 120 second, isolated bulges faded away and the etched dimple became superhydrophilic with a CA of 0° (Fig. 3k). High electric field at the edge region of the dimple polished this area and thereby destroyed the micro structures which were essential for superhydrophilicity. After 120 second etching, a crescent-like smooth area was generated at the edge of the etched dimple (Fig. 3d). Therefore, too high etching voltage is not appropriate for the second etching process.

**Figure 4:**
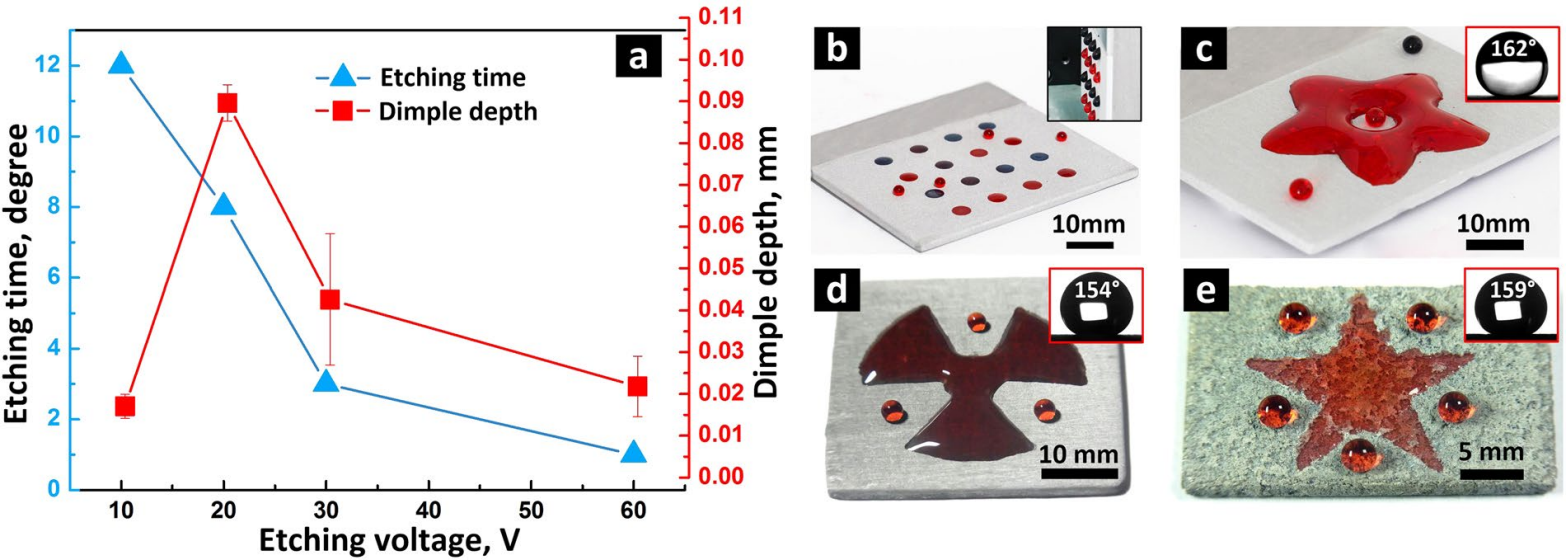
Depth of the dimples and photos of the fabricated superhydrophilic patterns. (**a**) Etching time and etched dimple depth for each etching voltage to obtain a dimple with CA lower than 20°. (**b**) Round superhydrophilic dimple array that was fabricated using the twice etching method; inset in (**b**) shows 20 μL droplets pinned by the dimple on the vertical substrate, diameters of the dimples are 4.0 mm. (**c**) Flower-like water trapped in the etched superhydrophilic dimple. Superhydrophilic patterns fabricated on (**d**) magnesium substrate and (**e**) titanium substrate using the twice etching method. Insets in (**c**–**e**) are CA images of superhydrophobic areas fabricated on aluminum, magnesium and titanium surfaces, respectively. For clear observation, the deionized water used for the demonstrations in (b-e) was dyed red or blue using food colouring.

Above all, the etching process in low voltage was not uniform and therefore took more time to remove the entire self-assembled hydrophobic layer on the surface (Fig. 4a). However, the dimple obtained at low etching potential cannot be too deep due to the limited current density (Fig. 4a). On the contrary, high voltage guarantees a more homogeneous etching process which results in a reduced etching time and less material removed from the entire superhydrophobic layer. That was why the depth of the obtained dimples with CAs below 20° decreased with the increase of etching voltage after 20 V. To obtain a uniform superhydrophilic dimple, a 60 V etching voltage could be applied first for ~1 minute to generate a homogeneous etched layer. To avoid destroying the micro structures at high voltage, the etching voltage can then be switched to 30 V for ~2 minutes to produce a flat superhydrophilic dimple. Figure 4b shows the photo of round superhydrophilic dimple array which was fabricated using the proposed etching step. The fabricated superhydrophilic dimple array can be employed as a water storage platform. The inset of Fig. 4b shows 20 μL droplets pinned firmly on the dimple array on a vertical superhydrophobic substrate. Superhydrophilic dimples with complex shapes, e.g. flower-like one, can also be produced when the corresponding mask is employed (Fig. 4c). The superhydrophobic area of the patterned surface had a CA as high as 162° ± 2.4 (inset of Fig. 4c) showing great wettability contrast with the etched superhydrophilic dimple area.

The twice electrochemical etching method is universal and can be extended to different metal substrates. Figure 4d and e show superhydrophilic dimples with different shapes that were fabricated on magnesium (Fig. 4d) and titanium substrates (Fig. 4e) using the proposed method. CAs of the surrounding superhydrophobic areas of magnesium and titanium patterned surface were 154° ± 3.8 and 159° ± 2.1, respectively (insets in Fig. 4d and e). The etching parameters for these two metal substrates can be found in the Supplementary Information.

### Fabrication of long-term superhydrophilic dimple patterns

**Figure 5:**
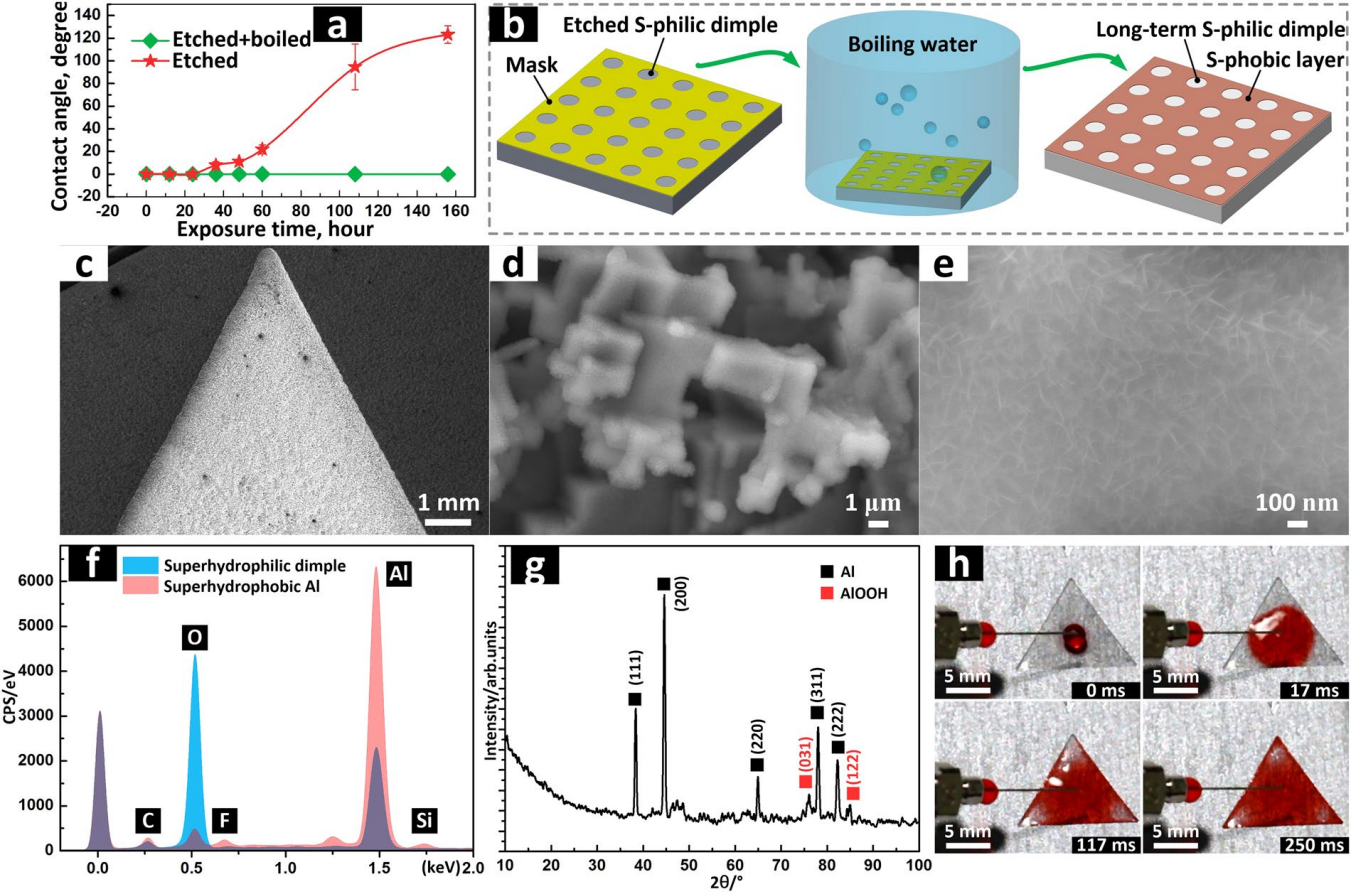
Fabrication of long-term superhydrophilic dimple patterns on the Al substrate. (**a**) CAs of the fabricated dimples versus the exposure time in air. (**b**) Schematic of the boiling-water immersion method. (**c**) SEM images of a long-term superhydrophilic triangle dimple that was fabricated by combining twice etching method and boiling-water immersion method. (**d**,**e**) Magnified SEM images of the area in the triangle dimple. (**f**) EDS spectrum of superhydrophobic Al and the fabricated long-term superhydrophilic dimple. (**g**) XRD spectrum of the fabricated long-term superhydrophilic dimple. (**h**) Sequence of images showing dyed droplet spreading rapidly in the superhydrophilic triangle dimple; the fabricated long-term triangle has a side length of 14 mm; the deionized water was dyed red (food colouring) for clear observation.

As reported in previous research, superhydrophilicity of many fabricated micro/nano structures can gradually transform to hydrophobicity or superhydrophobicity with an increase in exposure time in air^65–67^. The superhydrophilicity of the dimple which was electrochemically etched at 30 V in this work was only kept for 24 hours in air. The CA of this etched dimple increased rapidly to ~95° after air exposure for four and a half days (Fig. 5a). This wettability transition issue of the fabricated superhydrophilic patterns seriously hinders its applications in various domains, e.g. wettability transition can reduce the service life of superhydrophilic patterns for fog harvest applications. Hence, to solve the wettability transition problem the boiling-water immersion method^67^ was combined with the twice electrochemical-etching method to fabricate dimples with long-term superhydrophilicity.

As illustrated in Fig. 5b, the superhydrophobic substrate, which had been etched with a mask, was then immersed in boiling water for ~20 minutes. In this process, the etched dimple area was exposed to boiling water while the other area was protected from the boiling water by the mask. Next the mask was removed and a long-term superhydrophilic dimple with a shape that corresponded to the mask was finally obtained on the superhydrophobic substrate. As shown in Fig. 5c, a long-term superhydrophilic triangle dimple with a side length of 14 mm was fabricated on a superhydrophobic Al substrate. From Fig. 5c the boundary between the superhydrophobic substrate and boiled superhydrophilic dimple is clearly demarcated. Needle-like nano structures are distributed uniformly on step-like micro structures (Fig. 5d and e). EDS spectrum demonstrates that oxygen on the dimple significantly increased after boiling-water immersion process (Fig. 5f). The increased oxygen should be ascribed to boehmite (γ-AlOOH, Al_2_O_3_∙H_2_O) which was produced during the boiling-water immersion process. The diffraction peaks of AlOOH in the XRD spectrum further verifies this assumption (Fig. 5g). When a droplet touched the etched and boiled dimple, the droplet spread and covered the whole triangle dimple after ~250 ms. (Fig. 5h).

### Fog harvest process on the long-term superhydrophilic dimple patterns

It has been verified that a superhydrophobic-superhydrophilic patterned surface similar to the desert beetle can be applied for high-efficient fog harvest in the drought environment^20, 22, 23^. Dependence of pattern shapes^20, 68^ and patterns distribution^22, 23, 68^ on the fog harvest efficiency have been investigated in previous research. For instance, Megaridis *et al*.^68^ presented that optimal spatial nucleation, departing droplet size miniaturization and rapid drainage of condensate were three key factors to improve the dropwise condensation (DWC) performance. They proposed an interdigitated bioinspired surface which was composed of hydrophilic backgrounds and wedge-shaped superhydrophilic patterns. The wedge-shaped patterns have the capacity of pumpless transport of condensing liquid because of the Laplace pressure gradient^39^ and hence can facilitate the sustained condensate drainage.

**Figure 6:**
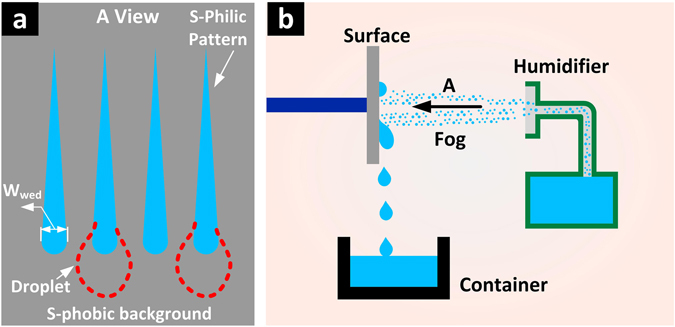
Schematic illustrations of fog harvest process on the fabricated patterned surface. (**a**) Top-view schematic of the superhydrophobic-superhydrophilic patterned surface. (**b**) Schematic of the fog harvest set. Dimension of the patterns in (**a**) was illustrated in Fig. S2a in the Supplementary Information.

In this section, the superhydrophobic surface with wedge-shaped superhydrophilic dimple array was fabricated using the optimized electrochemical etching method and boiling-water immersion method (Fig. 6a, see Supplementary Information Fig. S2a). The fog harvest process on the surface was carried out and compared with other patterned surfaces including superhydrophobic surface with round superhydrophilic dimple array (see Supplementary Information Fig. S2b), superhydrophobic surface with connected round superhydrophilic dimple array (see Supplementary Information Fig. S2c), bare superhydrophilic and superhydrophobic surfaces. Areas of these patterned superhydrophilic domains were made constant. The fog harvest process was illustrated in Fig. 6b. The fog harvest devices and measurements methods are described in the methods section.

**Figure 7:**
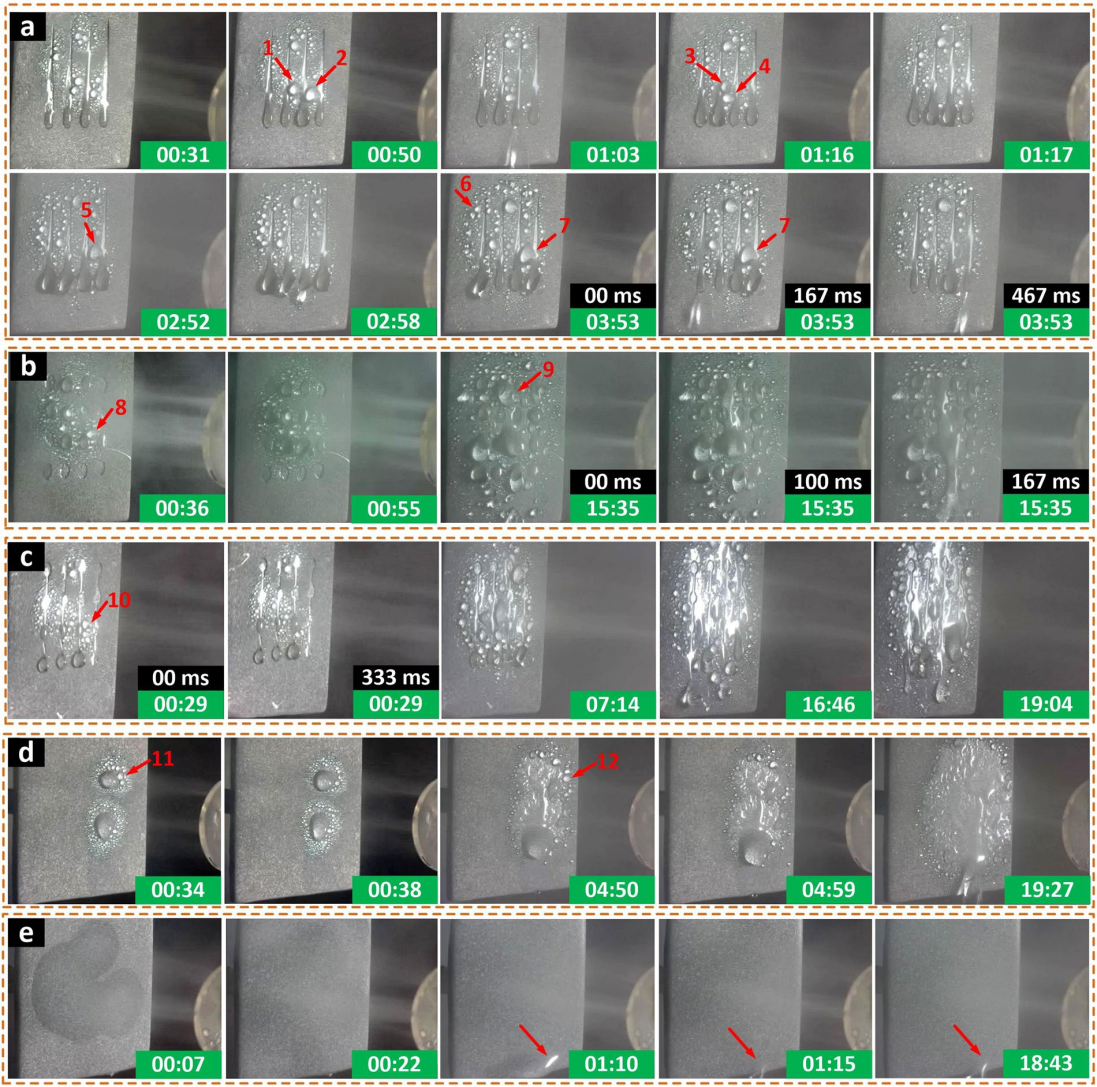
Fog harvest processes on different surfaces. Fog harvest processes that were carried out on the (**a**) superhydrophobic surface with wedge-shaped superhydrophilic dimple array, (**b**) superhydrophobic surface with round superhydrophilic dimple array, (**c**) superhydrophobic surface with connected round superhydrophilic dimple array, (**d**) bare superhydrophobic surface and (**e**) bare superhydrophilic surface. Dimension of the substrates was 40 × 40 mm. Positions 1–12 marked by red arrows in (**a**–**d**) were some selected positions where the nucleated droplets were transported into the tracks. Dimensions of the patterns in (**a**–**c**) were illustrated in Fig. S2a–c, respectively in the Supplementary Information.

The superhydrophilic area shows strong fog adsorption capacity; therefore, water condensed fast on the wedge-shaped dimples forming filmwise condensation (FWC) domains. Nonetheless, condensation of water on the existing film proceeded with a much lower rate compared with heterogeneous nucleation on the superhydrophobic background (DWC domains). Droplet nucleation and growth took place rapidly on the DWC domains. The nucleated droplet grew and would be transported into the FWC domains after touching superhydrophilic wedge-shaped tracks on one or both sides due to the internal Laplace pressure gradient of the liquid (selected positions 1–7 marked by arrows in Fig. 7a). Meanwhile, the liquid condensing on or transported into the track was pumped rapidly to the tail end of the track and subsequently shed off when growing beyond a threshold volume where the droplet weight exceeded the pining force at the tail end of the track. The superhydrophobic background in conjunction with the wedge-shaped tracks guaranteed high-efficient nucleation and rapid drainage of the formed condensate to leave pristine regions for a new cycle of nucleation and drainage (see Supplementary Information Movie S1). For example, nucleated droplets at positions 6 and 7 were sucked into tracks, then transported and merged into the condensate at the tail end of the track, which made the converged droplet depart. This superhydrophobic-superhydrophilic patterned surface showed a high water collection rate (WCR), harvesting ~12283 mg water in 1 hour.

On the superhydrophobic surface with round superhydrophilic dimple array, FWC and DWC domains were similarly formed rapidly on the superhydrophilic dimple and superhydrophobic background, respectively. Nucleated droplets on the DWC domain grew, merged and were transported into the condensing droplet on the dimple. Droplets pinned on dimples could not converge and therefore drainage of condensing liquid lagged. The DWC droplets, which could not shed off quickly, grew in size and merged forming large-area FWC domains (Fig. 7b, see Supplementary Information Movie S2). In other words, the delayed shedding of the condensing droplet facilitated gradual transition to FWC and resulted in a relatively low WCR of 9143 mg·h^−1^. If the round dimples were connected by superhydrophilic channels (see Supplementary Information Fig. S2c), the FWC droplets on dimples would flow through the channel under gravity and converge in droplets that were pinned on the lowest dimple. However, the converging rate was not enough to promote early drainage of condensing liquid. In addition, the DWC droplets were easy to merge and form large-area FWC domains because of the limited DWC area which was confined by two adjacent channels. As a sequence, the connected superhydrophilic dimple array actually led to a faster transition to FWC (Fig. 7c, see Supplementary Information Movie S3).

When the harvest process was implemented on the bare superhydrophobic surface, the DWC took place. However the depart droplet size on the DWC domains was too large to promote efficient drainage of condensing droplets because of the great pinning force on the wetted superhydrophobic surface. The size of condensing droplets grew; neighboring droplets merged and formed bigger droplets after making contact, which was similar to that on the superhydrophobic surface with round dimple array pattern, resulting in a fast transition to FWC (Fig. 7d, see Supplementary Information Movie S4). For the bare superhydrophilic surface, the fog condensed on the surface and spread rapidly to form a uniform FWC domain in only 22 s. However, water condensation on the film proceeded with low rate; water flowed downwards under gravity, forming a big water bump at the bottom of the substrate. The condensing water took more time to converge and shed off the surface due to absence of wettability patterns to provide a TPCL confine. As a sequence, the superhydrophilic surface had the lowest WCR of ~6373 mg·h^−1^ compared with other four surfaces (Fig. 7e, see Supplementary Information Movie S5).

**Figure 8:**
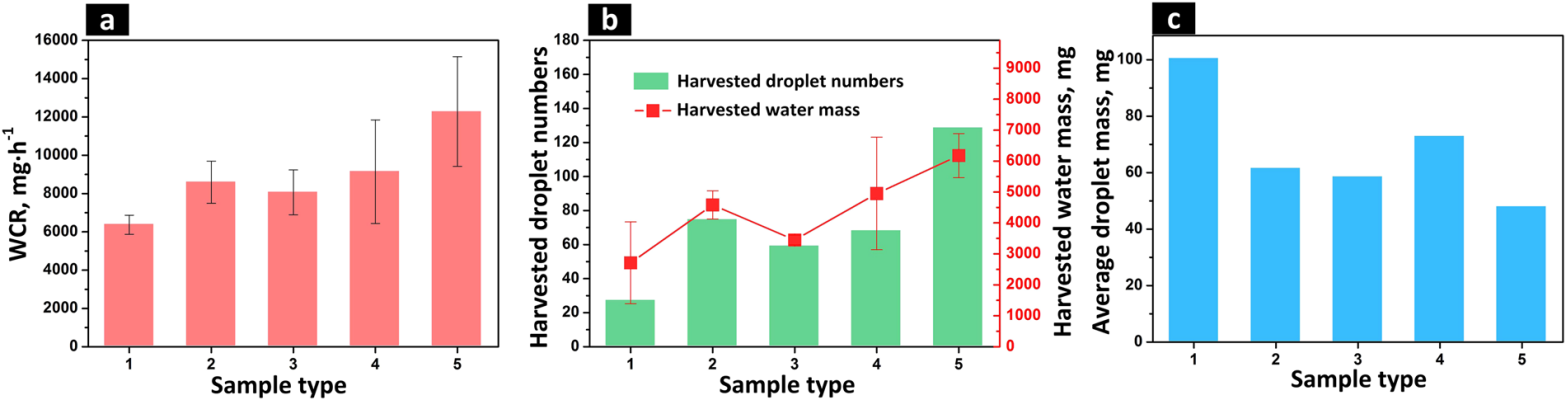
Fog harvest results on different surfaces. (**a**) Water collection rate of different surfaces in the fog harvest process. (**b**) Droplet number and total mass of water collected on different surfaces for 30 minute fog harvest process. (**c**) Average mass of each collected droplet in fog harvest processes on different surfaces. Surface types 1–5 were bare superhydrophilic surface, bare superhydrophobic surface, superhydrophobic surface with connected round superhydrophilic dimple array, superhydrophobic surface with round superhydrophilic dimple array and superhydrophobic surface with wedge-shaped superhydrophilic dimple array, respectively.

As shown in Fig. 8a, the WCR for wedge-shape patterned surface was highest, showing 92.7%, 43.0% and 34.4% improvement respectively when compared to bare superhydrophilic surface, bare superhydrophobic surface and round dimple array patterned surface (Fig. 8a). The wedge-shape patterned surface was capable of rapidly draining away condensate, having an average of 129 water drops shedding off the surface in 30 minutes (Fig. 8b) while the bare superhydrophilic surface, bare superhydrophobic surface, round dimple array patterned and connected round dimple array patterned surfaces only drained away 27, 75, 68 and 59 droplets, respectively from the surfaces in the same fog harvest process. Additionally, the wettability confine of the wedge-shaped superhydrophilic pattern reduced the departing droplet size. The volume with which the droplet may depart (departing droplet size) on the wedge-shape patterned surface can be predicted based on the Furmidge equation^69^,

1 $$\rho Vg\,\sin \,\alpha =\gamma \cdot {W}_{{\rm{wed}}}\cdot (\cos \,{\theta }_{{\rm{RD}}}-\,\cos \,{\theta }_{{\rm{AS}}})$$

where *ρ* is the density of harvested liquid, *V* is the departing droplet volume, *g* is the acceleration of gravity, *α* is the inclination of the substrate in the fog harvest process, *γ* is the surface tension of the liquid, *W*_wed_ is the width of the tail end of the wedge-shaped pattern (Fig. 6a), *θ*_RD_ and *θ*_AS_ are receding CA on the dimple and the advancing CA on the superhydrophobic background, respectively. In this work, the substrate was fixed vertically, so the departing droplet volume can be calculated using the following equation,

2 $$V=\gamma \cdot {W}_{{\rm{wed}}}\cdot (\cos \,{\theta }_{{\rm{RD}}}-\,\cos \,{\theta }_{{\rm{AS}}})/\rho g$$

Using water surface tension γ of 72.0 μN·mm^−1^, *W*_wed_ of 3.0 mm, the *θ*_RD_ and *θ*_AS_ of 42.5° and 166.2° ^35^, *ρ* of 1.0 g·cm^−3^ and the acceleration of gravity *g* of 9.8 N·kg^−1^, the departing volume on the wedge-shape patterned surface can be calculated to be ~37.3 μL, similar to the experimental average value of 47.9 μL (Fig. 8c) on the wedge-shaped pattern. According to Equation 2, different departing droplet volumes in the fog harvest process can be achieved by regulating the wedge-shaped pattern width. This research could possibly be applied in the design of new fog harvest devices that can meet all of the three key factors (i.e., optimal spatial nucleation, departing droplet size miniaturization and rapid drainage of condensate) to enhance the DWC performance.

## Conclusions

Electrochemical-etching, an environmental-friendly and inexpensive non-traditional machining method, was proposed to fabricate superhydrophilic dimple patterns directly on the etched superhydrophobic Al substrate. Superhydrophilic dimple patterns with different shapes can be obtained by directly etching the superhydrophobic Al substrate with masks. Superhydrophobic Al substrate showed high electrochemical corrosion potential due to the bubbles adhered on the surface. Therefore the applied potential in the electrochemical etching process must be high enough to conquer the electrochemical corrosion potential to uniformly remove the self-assembled hydrophobic layer of the superhydrophobic surface. Higher etching potential generates a more uniform etched layer, but long-time etching at high potential also results in problems like intense electric field which can destroy the micro structures. Hence, high etching voltage can be applied first to uniformly remove the hydrophobic layer. And to avoid destroying the micro structures, the etching potential should be switched to a relatively low value to produce a flat superhydrophilic dimple. By combining the electrochemical etching and boiling-water immersion methods, the superhydrophilicity of the fabricated dimples can be made long-term. The electrochemical-etching method is universal and can be extended to fabricate superhydrophilic patterns on various metal substrates like magnesium and titanium.

A bioinspired superhydrophobic surface with long-term wedge-shaped superhydrophilic patterns was fabricated using the optimized electrochemical-etching method. The fog harvest test shows that a superhydrophobic surface with wedge-shaped pattern array has high-efficient water collection rate because of rapid condensate drainage. In addition, the wettability contrast of the patterns produces a confined droplet-substrate interface for fast droplet departing. This easy-to-depart phenomenon can be well explained by the Furmidge equation. This research could be applied in the design of new fog harvest devices that can meet the three key factors (i.e., optimal spatial nucleation, departing droplet size miniaturization and rapid drainage of condensate) to enhance the dropwise condensation performance.

## Methods

### Fabrication of the superhydrophobic-superhydrophilic patterned substrate

6061 Al substrate (Suzhou Metal Material Manufacturer, China) was first electrochemically etched at 500 mA·cm^−2^ for 6 minutes in 0.1 mol·L^−1^ NaCl solution to obtain superhydrophilic micro structures. Then the etched surface was immersed in 1 wt% fluoroalkylsilane ethanol solution to decrease the surface energy. Polyimide tape with cutted hollowed-out patterns served as a mask and was firmly attached to the superhydrophobic surface. The masked surface was etched again at 10 V, 20 V, 30 V and 60 V for different times in a 0.1 mol·L^−1^ NaCl solution. After peeling off the tape, superhydrophobic surfaces with superhydrophilic patterns were obtained.

### Fog harvest measurements

The prepared surface was mounted on a sample holder in a glass box with a sliding roof (length × width × height: 500 × 300 × 500 mm). The sample was vertical to the horizontal plane and a culture dish was placed under the sample. Distance between the sample and nozzle of the fog generator was 40 mm. Simulated fog was generated by a household humidifier (Humidifying capacity: 300 ml·h^−1^, Power: 25 W). The collected water was dripped down into the dish under gravity and was weighted by a precision balance (Resolution: 0.1 mg) after 1 h harvest process.

### Characterization

A scanning electron microscope equipped with energy-dispersive X-ray spectroscope (SUPRA 55 SAPPHIRE, Germany) and an X-ray power diffractometer (Empyrean PANalytical, Holland) were employed to investigate the microtopography and chemical components of the fabricated surfaces. An electron microscope (MVVD030SC Microvision, China) was applied to capture the images of water droplets on the samples. Contact angle (CA), which is defined as the angle between the outline of solid-liquid interface and the tangent of liquid-vapor interface at the solid–liquid–vapor contact point^70^, was measured according to the sessile-drop method using an optical contact angle meter (DSA100, Krüss, Germany) and the final values were the averages of five measurements at five different locations. Depths of fabricated superhydrophilic patterns were measured using a coordinate measuring machine (ZEISS PRISMO, Germany). Electrochemical measurements were carried out in 1 wt% NaCl aqueous solution at ambient temperature using a potentiostat (Parstat 2273 Princeton, USA). For the polarization measurement, a three electrode configuration was used. A tested surface (circle area, 1 cm^2^), a platinum electrode and a saturated KCl-AgCl electrode served as the working electrode, the counter electrode and the reference electrode, respectively. The sweep rate was held at 0.5 mV·s^−1^ for all measurements.

### Data Availability

All data generated or analysed during this study are included in this published article (and its Supplementary Information files).

## Electronic supplementary material

Supplementary Information

Movie S1

Movie S2

Movie S3

Movie S4

Movie S5
