# Supplementary material for: A Twice Electrochemical-Etching Method to Fabricate Superhydrophobic-Superhydrophilic Patterns for Biomimetic Fog Harvest
Source: Sci Rep. 2017 Aug 18;7:8816. doi: 10.1038/s41598-017-09108-1 (PMC5562878; doi:10.1038/s41598-017-09108-1)
Supplement: Supplementary file 1 — Supplementary Information [file 41598_2017_9108_MOESM1_ESM.pdf]

Supplementary Information

A Twice Electrochemical-Etching Method to Fabricate Superhydrophobic-Superhydrophilic Patterns for Biomimetic Fog Harvest

Xiaolong Yang[†], Jinlong Song[†‡]*, Junkai Liu[†], Xin Liu[†], Zhuji Jin[†‡]

[†]Key Laboratory for Precision and Non-traditional Machining Technology of the Ministry of Education, Dalian University of Technology, Dalian 116024, P. R. China.

[‡]Collaborative Innovation Center of Major Machine Manufacturing in Liaoning, Dalian University of Technology, Dalian 116024, P. R. China.

*Corresponding author: Email: songjinlong@dlut.edu.cn (J.L Song), Tel: +86-411-84708422.

Fabrication of superhydrophilic dimple patterns on magnesium surface

Magnesium substrate (Suzhou Metal Material Manufacturer, China) was polished and firstly electrochemically etched at 450 mA·cm^{-2} in 0.2 mol·L^{-1} NaCl solution for 6 min to obtain superhydrophilic rough structures.[1] Then the etched surface was immersed in 1wt% fluoroalkylsilane ethanol solution for 60 min. Polyimide tape mask was firmly attached on the superhydrophobic substrate. The masked substrate was second etched at 10V in the 0.2 mol·L^{-1} NaCl solution for ~4 min. After the tape mask was peeled off, superhydrophobic substrates with superhydrophilic dimple patterns were finally obtained.

Fabrication of superhydrophilic dimple patterns on titanium surface

Titanium substrate (Suzhou Metal Material Manufacturer, China) was polished and electrochemically etched at 1000 mA·cm^{-2} in 0.2 mol·L^{-1} NaCl solution for 8min to obtain superhydrophilic rough structures.[2] Then the etched surface was modified for 60 min in 1wt% fluoroalkylsilane ethanol solution. The masked substrate was second etched at 60V for 1 min and subsequently at 30 V for 2 min in the 0.2 mol·L^{-1} NaCl solution to obtain corresponding superhydrophilic dimple patterns.

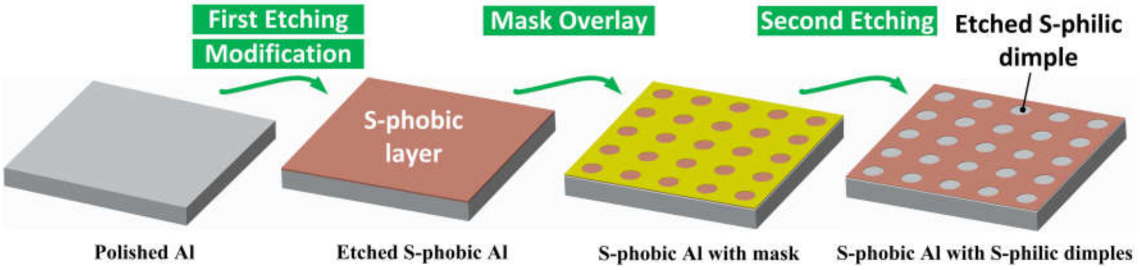

Figure S1. Schematic illustration of fabricating superhydrophilic dimples using a twice electrochemical-etching method.

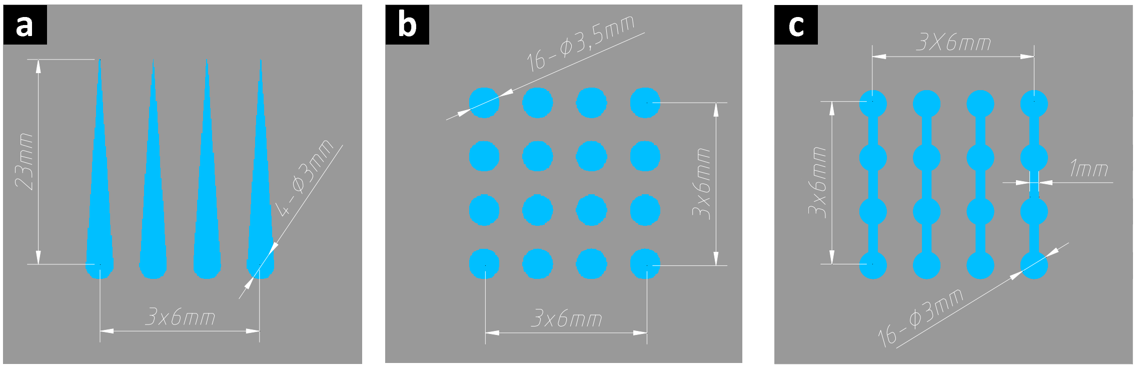

Figure S2. Dimensions of the patterned surfaces. (a) Dimension of the superhydrophobic surface with wedge-shaped superhydrophilic dimple array. (b) Dimension of the superhydrophobic surface with round superhydrophilic dimple array. (c) Dimension of the superhydrophobic surface with connected round superhydrophilic dimple array. Dimensions of the substrates were 40×40 mm; the patterned area of these three surfaces was ~150 mm^2

References:

1. Xu, W., Song, J., Sun, J., Lu, Y. & Yu, Z. Rapid fabrication of large-area, corrosion-resistant superhydrophobic Mg alloy surfaces. *ACS Appl. Mater. Interfaces.* **3**, 4404-4414 (2011).

2. Lu, Y. et al. Preparation of superhydrophobic titanium surfaces via electrochemical etching and fluorosilane modification. *Appl. Surf. Sci.* **263**, 297-301 (2012).
